# Utilization of qPCR to Determine Duration and Environmental Drivers Contributing to the Persistence of Human DNA in Soil

**DOI:** 10.3390/genes15060741

**Published:** 2024-06-05

**Authors:** Hannah L. Noel, Rebecca L. George, Brittania Bintz, Maureen Peters Hickman, Frankie West

**Affiliations:** 1 Department of Microbiology, University of Tennessee, Knoxville, TN 37996, USA; 2 Department of Anthropology and Sociology, Western Carolina University, Cullowhee, NC 29723, USA; rgeorge@wcu.edu; 3 Department of Chemistry and Physics, Forensic Science Program, Western Carolina University, Cullowhee, NC 29723, USA; bbintz@wcu.edu; 4 Department of Biology, Western Carolina University, Cullowhee, NC 29723, USA; mphickman@wcu.edu

**Keywords:** soil taphonomy, DNA degradation, quantitative PCR, DNA persistence, human decomposition

## Abstract

Little is known about the underlying mechanisms that contribute to the persistence and degradation of DNA within soil. The goals of this study are to determine the duration of mitochondrial DNA (mtDNA) and nuclear DNA (nuDNA) persistence in soils enriched by surface-level human decomposition and to better understand the contribution of environmental factors. The surface-level decomposition of three human cadavers was documented over 11 weeks. Based on quantitative PCR results, we found nuDNA to persist in soils six weeks post-placement, while mtDNA was recoverable for the entire 11-week decomposition period. Principle components analyses and Spearman’s rank correlations revealed that (1) time, (2) total body score, and (3) weekly average air temperature were significantly correlated with concentrations of nuDNA and mtDNA in soil, suggesting these factors play a role in the degradation of DNA in soils.

## 1. Introduction

During decomposition, human remains are subjected to various environmental factors that can influence taphonomic changes and decomposition rates. Along with anatomical remains, biological evidence in the form of DNA will also degrade over time, a process that is heavily influenced by the surrounding environment. In cases involving clandestine burials and outdoor surface-level decomposition, DNA found in decomposition fluids interacts directly with the soil matrix. Within this interface, cells autolyze via hydrolytic enzymes [1] and DNA becomes extracellular. Nuclease enzymes from decomposition fluids and soil microbes cleave double-stranded nuclear DNA, causing fragmentation and the loss of genetic information [2,3]. Furthermore, extracellular DNA devoid of intracellular repair systems is prone to double-stranded breaks facilitated by base pair oxidation [4].

However, there is evidence that mammalian DNA may persist in soil and sediment matrices for extended periods [5,6,7,8,9]. Under specific circumstances, ancient DNA from Neanderthals and Denisovans has remained resistant to degradation and yielded analyzable mitochondrial DNA (mtDNA) sequence data [7,8]. Similarly, a contemporary study of surface-level decomposition by Emmons et al. provided evidence that quantifiable amounts of mtDNA were generally more recoverable from soils and persisted longer into the decomposition process than nuclear DNA (nuDNA) [9]. Though the circular structure and high copy number per cell of mtDNA likely contribute to its resilience in comparison to nuDNA, the surrounding environment also plays a role. For instance, chemical factors in the soil such as free ions and humic acid can create cation bridges with the negatively charged sugar-phosphate backbone of DNA, allowing it to remain bound in the soil matrix [2,10]. Soil types with high clay content may also be beneficial to DNA persistence as the low porosity and small particle size allow for an increased surface area to form ionic bonds, lend to micro-aggregation, and reduce oxidizing processes [5,11,12].

Along with the chemical and physical properties of soil, climate may also have an impact on the degradation of DNA in soils, especially at the interface between the soil surface and decomposition fluids. Typically, the putrefaction of both human remains and pig carcasses occurs optimally at warm temperatures [1,13,14]. Additionally, moisture from humidity in surface-level human decomposition studies [13] and increased soil moisture in mouse burial models [15] have been characterized as factors that increase the rate of decomposition. Both warmer temperatures [14] and increased levels of moisture [15] in decomposition contexts can be linked to greater microbial diversity and activity in soils. Under these conditions, DNA may be more prone to degradation from extracellular nucleases secreted by some bacteria.

To better understand the role of climate in the persistence of DNA in soil, this research aims to expand upon Emmons et al. by monitoring the concentration of mtDNA and nuDNA in soil following surface-level decomposition in conjunction with temperature, humidity, rainfall, and body moisture levels. As soil can act as a natural reservoir for storing biological evidence in the form of DNA, understanding the dynamics underlying the persistence of DNA in soil has the potential to support forensic investigations and anthropological research. This will be especially useful in cases of clandestine burials in which remains have been moved to a secondary location. This pilot study is meant to encourage further research on the persistence of extracellular human DNA in soil by elucidating some potential relationships with climatic variables.

## 2. Materials and Methods

### 2.1. Donors, Sampling Location, and Sampling Collection

This study was conducted at Western Carolina University’s Forensic Osteology Research Station (FOREST), an outdoor human decomposition facility located in Cullowhee, North Carolina. Three cadaveric donors, referred to, respectively, as Donors 1, 2, and 3, were the subjects utilized in this study. The three donors were from WCU’s willed body donation program and had consented to post-mortem DNA testing. All donors were elderly, of self-identified European ancestry, and died of natural causes. The reported sex of Donor 1 was female, while Donors 2 and 3 were reported male. Reported weight for donors upon intake were as follows: Donor 1 43 kg, Donor 2 89 kg, and Donor 3 41 kg. Donor 1 had wounds from a feeding tube port on their lower left abdomen and an open sore on their right lower jawline. These antemortem wounds were noted as they may attract scavenger activity early into placement [16]. Though the donors were placed within a fenced enclosure, black vultures and possums are common scavengers at the FOREST and were thus monitored. Donors were maintained in temperature-controlled storage before arrival at the FOREST.

Donors were placed in the surface-level decomposition enclosure at the FOREST. This enclosure is approximately 465 square meters and sloped, with the top of the slope being northeast, with partial canopy coverage. The enclosure is surrounded by a wooden privacy fence and a chain-linked fence topped with razor wire. Each donor was unclothed on the ground and placed in a supine position with their mouths open. The positioning of arms varied between donors. Donor 1 had arms crossed over their torso, Donor 2 had arms resting at their sides, and Donor 3’s hands rested on their hips. No scavenger barriers were placed. The donors were placed in three locations within the facility on different dates within the month of May. Donor 1 was placed at the top of the slope in the facility. Donor 2 was placed midway down the slope, roughly a meter away from the mummified remains of another donor. Donor 3 was placed at the bottom of the slope to the right of the facility entrance. Trail cameras were set at the foot of each body to monitor scavenging activity.

Soil samples were taken prior to placement and then weekly, for eleven weeks. At eleven weeks, donors reached complete skeletonization and were recovered from the FOREST. Weekly soil samples were taken from four locations around the remains: the left of the cranium (C), either side of the torso (right TR and left TL), and the right lower extremity (LE). All samples were taken from within an 8 cm distance from the remains. The soil was collected using Bel-Art Sterileware^®^ Sampling Spatulas (Bel-Art Products, Wayne, NJ, USA) and 50 mL conical tubes that had been sterilized via UV for 30 min before use. Enough soil was collected to reach the 10 mL marking on the 50 mL conical tubes. Organic matter and leaf litter were removed from the top layer of soil prior to collection. Each tube was sealed using parafilm and stored at −80 °C. The positioning of Donor 1 was greatly affected by scavenging from black vultures, causing the upper body and cranium to migrate below the lower extremities. To accommodate this positionality, samples from weeks four through eleven were taken from general regions of the plot: one upslope towards the top of the plot, one from each side of the center of the plot, and one downslope towards the bottom of the plot.

Along with weekly soil samples, total body score (TBS) and accumulated degree days (ADD) were determined using methods described by Megyesi et al. [17]. TBS utilizes a scoring metric assigned to key stages within the four broad categories of decomposition: fresh, early decomposition, advanced decomposition, and skeletonization. These stages are scored across three body regions: the head and neck, the trunk of the body, and the limbs. The scores of all three body regions are summed to produce the TBS. Fresh remains would be assigned a TBS of 3, while remains with greater TBS values would be considered more advanced in decomposition. ADD is a measurement of thermal units that encourage biological processes. This can be predicted from TBS using the following equation proposed by Megyesi et al. [17]:

(1) $$ADD=10^{\left(0.002*TBS*TBS+1.81\right)}\pm 388.16$$

### 2.2. Weather and Environmental Data Collection

Daily temperature, humidity, and precipitation measurements were recorded using a National Oceanic and Atmospheric Administration (NOAA) weather station located at Jackson County Airport, Airport Road, Sylva, North Carolina 28779, which is located within a two-mile radius of the FOREST. These data were converted to weekly averages to correspond to weekly soil sampling. Moisture retained by the donors was measured weekly using a Delmhorst^®^ RDM-3 moisture meter (Delmhorst Instrument Co., Towaco, NJ, USA) with default settings. Measurements were taken from the flesh of the right and left femora and sternum then averaged. Since the electrodes accurately detect 5% to 60% moisture, all readings that were >60% were converted to 60 for statistical analyses and considered fully saturated. Donor 1 had no flesh remaining on the left femur by week seven into the decomposition period, so body moisture averages were calculated from the right femur and sternum only. By week ten, Donor 1 entered advanced decay with no flesh present on any of the three sampling points. Therefore, no moisture measurements were taken for weeks ten and eleven from Donor 1.

### 2.3. DNA Extraction and Quantitation

DNA was extracted from 250 mg of soil using the QIAGEN DNeasy^®^ PowerSoil^®^ Pro kit (Hilden, Germany) following manufacturer protocols, including a 15 min bead-beating step using a horizontal vortex adapter. A blank extraction control was included with each extraction. Samples were eluted to a final volume of 50 µL and stored at −80 °C for downstream analysis. For the eleventh week of decomposition, only one soil sample was selected per donor for extraction, due to extraction reagents being limited. These samples were selected as they would likely contain relatively high concentrations of DNA based on trends in quantitation results for prior weeks. The samples selected were Donor 1 TL-11, Donor 2 TL-11, and Donor 3 C-11.

Human nuDNA quantification was performed using the Applied Biosystems Quantifiler™ Trio DNA Quantification Kit (Waltham, MA, USA) and analyzed on the Applied Biosystems™ 7500 Real-Time PCR system (Waltham, MA, USA). This commercially available assay allows for simultaneous quantification of a small autosomal (SA) target, a large autosomal (LA) target, a Y-chromosomal target, and an internal positive control (IPC) and enables assessment of DNA degradation and inhibition. Degradation indices (DI) were calculated by dividing the concentration of the SA target (80 bp) by the concentration of the LA target (214 bp), as per manufacturer protocols [18]. A DI ≤ 1 indicates intact DNA, while a DI > 1 indicates degradation.

Human mtDNA quantification was performed using a triplex quantitative PCR (qPCR) assay described by Kavlick [19] and run on the Applied Biosystems™ 7500 Real-Time PCR system (Waltham, MA, USA). This triplex assay consists of a short (105 bp) mtDNA target in the ND5 Gene, a long (316 bp) mtDNA target in the 16S rRNA Gene, and an IPC [19]. Degradation of mtDNA was assessed by calculating ΔΔCt value using the following equation:

(2) $$\Delta \Delta Ct={\Delta Ct}_{Sample}-{\Delta Ct}_{Calibrator\;}={\left({Ct}_{316}-{Ct}_{105}\right)}_{Sample}-{\left({Ct}_{316}-{Ct}_{105}\right)}_{Calibrator}$$

where Ct_316_ is the cycle threshold of the long target and Ct_105_ is the cycle threshold of the short target for both the sample DNA and the HL60 calibrator positive control DNA. A ΔΔCt value of zero is interpreted to be undegraded, a ΔΔCt value of one is interpreted as slight degradation since there are half as many large target copies as the small target, and ΔΔCt values of four suggest 24 fewer large targets than small, indicating a higher state of sample degradation [19]. Three technical replicates were used for each sample along with a non-template control for both nuDNA and mtDNA quantification methods.

### 2.4. Statistical Analysis

Donors (*n* = 3) were treated as experimental replicates. A principal component analysis (PCA) of the environmental and weather data, along with time into decomposition period, was performed. An ANOVA was used to compare the quantity of DNA to each dimension of the PCA, which were visualized as linear models. Spearman’s rank correlation, or Spearman’s ρ, was calculated to determine the degree of association between individual environmental variables and sample quantity. Due to differences in weight and physical placements of donors, the Friedman’s and Wilcoxon signed-rank tests were used to determine if a statistically significant difference existed between weekly TBS for donors. Statistical analyses and visualizations were performed in R Statistical Software v.4.3.2 (31 October 2023) using dplyr, FactoMineR, factoextra, ggplot2, kableExtra, and stats packages [20,21,22,23,24,25].

## 3. Results

### 3.1. NuDNA Quantitation and Persistence

**Figure 1:**
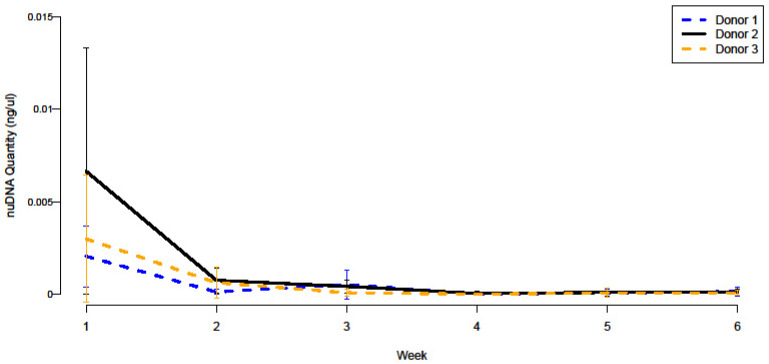
The average concentration of small autosomal target (80 bp) detected in soil samples taken from the plots of three donors over six weeks of decomposition. Standard deviation was calculated using the replicate quantitation of the four weekly samples per donor and is represented with error bars.

On average, the quantity of nuDNA detected did not exceed the limit of detection. NuDNA was detected in subcellular quantities during the first six weeks of the decomposition period for all three donors, decreasing over time as shown in Figure 1. DNA concentrations for the SA target were typically higher than the concentrations reported for the LA target (Table 1), indicating that the nuDNA recovered was highly degraded. Y-chromosomal DNA was recovered during the first two weeks of decomposition from soils surrounding Donors 2 and 3, matching the donor’s self-reported biological sex (Table 1).

The weekly data points are averages of the three technical replicates of each of the four sampling locations per donor (Supplemental Tables S1–S3). Due to the nature of qPCR and its sensitivity to spurious contamination and pipetting error, some replicate wells were removed from quantitation calculations. Outliers were removed based on the Ct standard deviation (Ct-SD), similar to the protocol outlined in Maussion et al. [26]. If the Ct-SD was greater than 0.3, then the replicate furthest from the sample mean was removed from the calculations. Highly variable replicates were preserved if the absolute (mean − median)/median was less than 0.1, as no clear outlier could be determined. This protocol was also applied to the interpretation of the mtDNA data presented in Section 3.3.

### 3.2. NuDNA and Environmental Drivers

**Figure 2:**
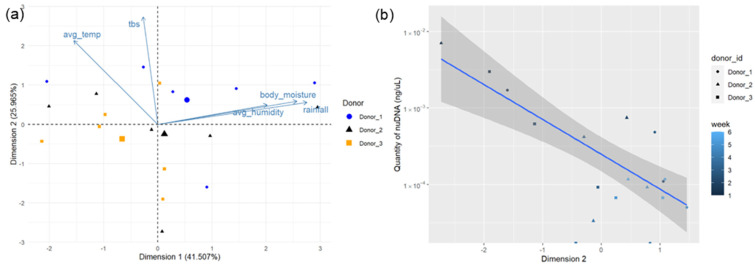
(**a**) PCA biplot of dimensions one and two for the first six weeks of data collected from decomposition of three human donors with environmental factors indicated with vectors. (**b**) Linear regression of concentration of nuDNA in weekly soil samples compared to dimension two of the PCA (*p*-value = 9.696 × 10^−5^). The *y*-axis has been converted to a logarithmic scale; grey shading represents the 95% confidence interval.

All data for the statistical analysis of nuDNA and environmental factors can be found in Supplemental Table S4. The PCA of environmental data for the first six weeks of decomposition showed no distinct clustering of data points (Figure 2a). Components one and two of the PCA explain 66.772% of the total variation within the data (41.507% and 25.965%, respectively, Supplemental Table S5). The variation in dimension one was positively correlated with the average weekly rainfall, body moisture content, and humidity, while dimension two was positively correlated to TBS and the weekly average temperature (correlation coefficients shown in Supplemental Table S6). Linear regressions comparing the dimensions of the PCA in Figure 2a to the quantity of nuDNA in the soil in ng/µL were analyzed with an ANOVA. A statistically significant negative correlation exists between dimension two of the PCA and the quantity of nuDNA in the soil (*p*-value = 9.696 × 10^−5^; Figure 2b). This indicates that TBS and the weekly average temperature have a negative impact on the quantity of nuDNA in soil over time. This is further supported by Spearman’s rank correlations performed between environmental variables and the quantity of nuDNA as shown in Table 2. There are statistically significant negative correlations between the number of weeks the remains decomposed and nuDNA concentration (Spearman’s ρ = −0.6594, *p*-value = 0.0029), the TBS of remains and nuDNA concentration (Spearman’s ρ = −0.4977, *p*-value = 0.0356), and the average weekly temperature and nuDNA concentration (Spearman’s ρ = −0.6184, *p*-value = 0.0062).

### 3.3. MtDNA Quantitation and Persistence

**Figure 3:**
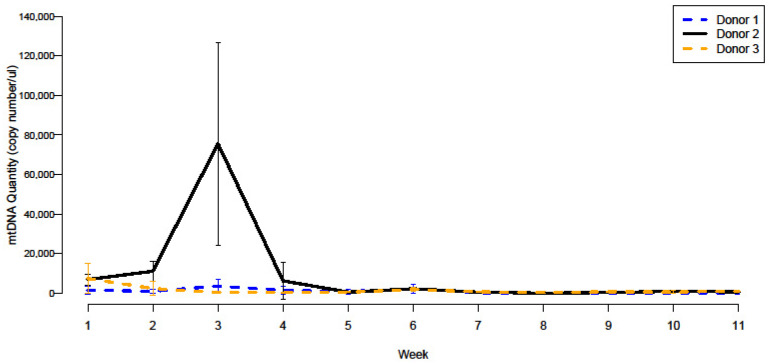
The average quantity of the short (105 bp) mtDNA target amplified from soil samples taken from the plots of three human donors over eleven weeks of decomposition. Standard deviation was calculated using the replicate quantitation of each of the four weekly samples per donor and is shown as error bars.

**Figure 4:**
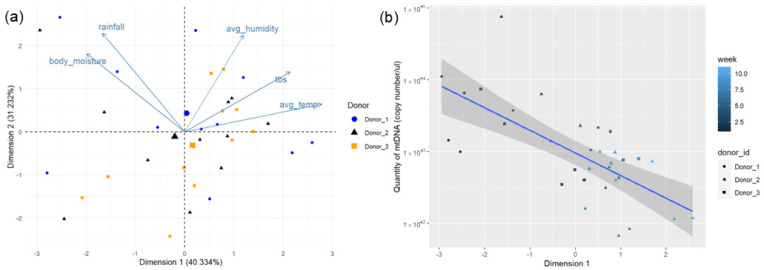
(**a**) PCA biplot of dimensions one and two for eleven weeks of data collected from the decomposition of three human donors with environmental factors indicated with vectors. (**b**) Linear regression of concentration of mtDNA in weekly soil samples compared to dimension two of the PCA (*p*-value = 6.177 × 10^−2^). The *y*-axis has been converted to a logarithmic scale; grey shading represents the 95% confidence interval.

MtDNA was detected from all three donors for the entire eleven-week decomposition period, persisting longer than nuDNA in soils. All quantitation data for averages can be found in Supplemental Tables S7–S9. The average quantity of the short (105 bp) mtDNA target detected for each donor per week is presented in Figure 3, showing the gradual decrease in quantity over time. ΔΔCt values generally increase over time, indicating the degradation of DNA (Table 3), similar to the increased DI values found in nuDNA (Table 1). Soils surrounding Donor 2 consistently had the highest abundance of both mtDNA and nuDNA (Figure 1 and Figure 4) during the first four weeks of decomposition. This may be due to intrinsic factors, such as Donor 2 having a higher body mass than both Donors 1 and 3. These data are averages of the technical replicates of each of the four weekly sampling locations per donor per week (Supplemental Tables S7–S9). No significant inhibition was detected, as there were relatively similar values between the IPC Ct of the standards and controls in comparison to experimental samples.

### 3.4. MtDNA and Environmental Drivers

All data for the statistical analysis of mtDNA and environmental factors can be found in Supplemental Table S10. The PCA of the environmental data for the full eleven-week decomposition period did not show any significant clustering of data points (Figure 4a). Components one and two of the PCA explain 71.566% of the total variation within the data (41.334% and 31.232%, respectively, Supplemental Table S11). The two variables that explain the majority of the variation in dimension one are weekly average temperature and TBS. For dimension two, most of the variation is explained by the weekly total rainfall and humidity, suggesting a moisture gradient (correlation coefficients shown in Supplemental Table S12). Linear regressions comparing the dimensions of the PCA in Figure 4a to the quantity of mtDNA in the soil in copy number/µL were analyzed with an ANOVA, showing no statistically significant correlations. However, the comparison of mtDNA quantity and dimension one has a *p*-value of 0.06177, which is close to the α value of 0.05. This suggests that there may be a potential negative correlation between dimension one of the PCA and the quantity of mtDNA, as shown in Figure 4b. This would suggest a potential relationship between TBS and the weekly average temperature on the quantity of mtDNA in soil over time, similar to trends seen between environmental factors and nuDNA quantity. This potential correlation is also supported by Spearman’s rank correlations performed between environmental variables and the quantity of nuDNA as shown in Table 4. There are statistically negative correlations between the mtDNA copy number and the number of weeks into the decomposition period (Spearman’s ρ = −0.6219, *p*-value = 0.0001), TBS of remains (Spearman’s ρ = −0.4687, *p*-value = 0.0059), average weekly humidity (Spearman’s ρ = −0.3439, *p*-value = 0.0500), and average weekly temperature (Spearman’s ρ = −0.7200, *p*-value = 2.3245 × 10^−6^). These trends are similar to the degradation of nuDNA (Table 3). There was a significant difference in decomposition rates between donors (Freidman’s Χ^2^ = 19.4, df = 2, *p*-value = 6.128 × 10^−5^). The resulting Wilcoxon rank sum test revealed significant differences in the weekly TBS between Donors 1 and 2 (*p*-value = 0.005) and Donors 1 and 3 (*p*-value = 0.002), but not between Donors 2 and 3 (*p*-value = 0.473).

## 4. Discussion

### 4.1. Recovery of DNA from Soil

Human mtDNA persisted for the entire eleven-week decomposition period, while nuDNA was largely below the limit of detection and completely unrecoverable after six weeks, similar to the findings presented by Emmons et al. [9]. The persistence of mtDNA is likely attributed to its robust circular structure causing it to be more resistant to nuclease activity and its high copy number per cell relative to nuDNA. The quantities of both nuDNA and mtDNA generally decreased over time, with only shorter targets detectable later into the decomposition period (Table 1 and Table 2). Both the Quantifiler™ Trio DNA Quantification Kit and custom mtDNA triplex assay contain short and long targets: 80 and 214 bp in Quantifiler Trio kit and 105 and 316 bp in the mtDNA triplex assay [18,19]. The preferential recovery of these “small” DNA targets may be due to double-stranded breaks from nuclease activity and oxidation, which fragment the DNA in the soil. Fragmentation resulting from the nuclease activity is reflected in the nuDNA DI and mtDNA ΔΔCt values (Table 1 and Table 2).

DNA quantity and degradation fluctuated over the decomposition period. For example, nuDNA was undetectable in week four for Donor 3, but 6.67 × 10^−5^ ng/µL of the SA target was detected for the same donor the following week (Table 1). These fluctuations may be due to the patterns of precipitation (Supplemental Tables S4 and S10). During advanced decomposition, the remains dry and purge fluids diminish [17]. However, it was observed that the remains in this study absorbed moisture after rainfall, causing fluctuations in the TBS as well as allowing for additional fluids to seep into the soil after the initial purge. This occurrence could potentially lead to the shedding of more genetic material into the soil over time, explaining the fluctuations in qPCR quantities. This phenomenon, during which remains are rehydrated with precipitation, has been documented in decomposition studies of swine in temperate South Africa, where the remains were noted to have absorbed moisture, leading to the release of more fluids and reinvigorated insect activity [27]. However, fluctuation could also be due to stochastic sampling since these results reflect the detection of subcellular levels of nuDNA. This trend of precipitation rehydrating remains may also account for the sudden increase in detected mtDNA for Donor 2 between weeks two and four of the decomposition period (Figure 3). During weeks two, three, and four, Donor 2 was exposed to 7.3406 cm, 5.5880 cm, and 1.2954 cm of rain, respectively (Supplemental Table S10). However, as the decomposition period progressed, weekly precipitation decreased, leading to the remains drying out and purging less fluids.

Notably, soils surrounding Donor 2 consistently contained higher quantities of both nuDNA and mtDNA than Donors 1 and 3 during the first four weeks of decomposition. Donor 2 weighed more than double the recorded weights of Donors 1 and 3. Body mass is an intrinsic factor that is thought to influence decomposition rates, though different studies have shown variable effects. Mann et al. report that an increase in body mass is associated with an increase in the rate of decomposition of remains due to the rapid liquefaction of body fat over muscle tissues, as observed in human decomposition studies performed at the Anthropology Research Facility at The University of Tennessee, Knoxville [28]. Conversely, Sutherland et al. report that increased body mass slows the rate of decomposition due to a plateau phase as seen in a comparative study of pig carcasses at the Forensic Anthropology Body Farm at the University of Pretoria, South Africa [27]. In this study, there was a significant difference in the decomposition rate of Donor 1 in comparison to Donors 2 and 3, but not between Donors 2 and 3. This was likely due to the rapid skeletonization of Donor 1 due to excessive scavenging activity from black vultures. Though no differences in decomposition rates seemingly exist regarding mass, the liquefaction of body fat could potentially result in increased amounts of purged fluids in the soil, resulting in the recovery of higher quantities of DNA. Furthermore, similar trends in nuDNA and mtDNA recovery can be seen between Donors 1 and 3 (Figure 1 and Figure 3). These two donors had similar weights upon intake. Future studies should consider donor intrinsic factors such as body mass in relation to the persistence of DNA in soils.

### 4.2. Effects of Environmental Variables on DNA Persistence

Statistical analysis suggests that the temperature, TBS, and duration of exposure to the external environment all have a significant impact on the persistence of human DNA in soil. High temperature is known to increase the rates of microbial activity, and, in turn, decomposition rates [1,14,29]. The quantities of nuDNA and mtDNA are expected to deplete as the remains transition through the phases of decomposition [9]. Spearman’s rank test correlations also revealed a statistically significant correlation between average weekly humidity and quantity of mtDNA (*p*-value = 0.0500, Table 4), but not with the quantity of nuDNA (*p*-value = 0.9477, Table 2). However, it is believed that moisture likely does play a role in the persistence of human DNA in soils, as it impacts decomposition rates. Typically, increased soil moisture in burial contexts promotes microbial activity [30] and can increase the rate of decomposition [1], so long as the soil’s matric potential to retain water is not exceeded [15]. Furthermore, temperature and moisture also likely play a nuanced role in the enzymatic activity of extracellular nucleases that contribute to DNA degradation. Many observations fall outside of the 95% confidence interval of the linear regressions in Figure 2b and Figure 4b. In Figure 2b, these observations do not appear to follow a trend concerning donors or weeks into the decomposition period. Similarly, in Figure 4b, observations that fall outside of this interval are not attributed to one donor or timepoint, though the furthest outlier is from Donor 2. This further conveys the broad variation between donors and the environmental factors influencing their decomposition.

Our study is limited to a small number of biological replicates and data points. Future studies need to be performed in different regions across multiple seasons to build predictive models on the recoverability of human DNA from soil. Additionally, the inclusion of soil edaphic factors such as pH, soil moisture, and electrical conductivity would help give better insights into the mechanisms underlying DNA’s persistence in soils. Furthermore, the varied placement of donors may have influenced the rate of decomposition and persistence of DNA in soils. Due to the sloped nature of the FOREST, Donors 1 and 2 were placed uphill while Donor 3 was placed downhill. During rainfall, precipitation could have caused runoff of decomposition fluids, along with DNA. Additionally, the sloped terrain may also cause differences in sun exposure on remains placed uphill rather than downhill, contributing to variations in decomposition rates. Future considerations to donor placement should ensure donors are on even terrain with similar light exposure to reduce these spatial influences.

## 5. Conclusions

Both human nuDNA and mtDNA have been shown to persist in soils disturbed by surface-level decomposition for weeks at a time, with mtDNA lasting for a longer duration. Therefore, the recovery of human mtDNA from soils may be a potential biomarker of human decomposition for forensic investigations. The development of standard methods for the recovery, storage, extraction, and sequencing of mtDNA recovered from soils could also be beneficial in medicolegal contexts where remains have been moved to a secondary location. Though temperature, TBS, and time into the decomposition period seem to have an impact on the degradation of DNA in soils, the nuanced mechanisms driving DNA persistence and degradation have yet to be elucidated. Further research on environmental and cadaveric donor intrinsic factors, in relation to the recovery of human DNA in soils, is needed to better understand these processes to benefit paleoanthropological and forensic investigation.

## Figures and Tables

**Table 1:** Quantification results for nuDNA from experimental samples including small autosomal target, large autosomal target, and Y-Chromosomal target DNA in nanograms per microliter.

Donor	Week	Small Target (ng/µL)	Large Target (ng/µL)	Y Target (ng/µL)	Degradation Index
1	1	1.71 × 10^−3^	3.27 × 10^−4^	0	5.22
	2	1.09 × 10^−4^	9.09 × 10^−6^	0	12.00
	3	4.82 × 10^−4^	0	0	*
	4	0	3.33 × 10^−5^	0	0.00
	5	5.00 × 10^−5^	0	0	*
	6	1.17 × 10^−4^	5.00 × 10^−5^	0	2.33
2	1	7.07 × 10^−3^	3.74 × 10^−3^	6.91 × 10^−3^	1.89
	2	7.45 × 10^−4^	1.18 × 10^−4^	3.27 × 10^−4^	6.31
	3	4.17 × 10^−4^	0	0	*
	4	3.33 × 10^−5^	8.33 × 10^−5^	0	0.40
	5	9.17 × 10^−5^	0	0	*
	6	1.17 × 10^−4^	1.67 × 10^−5^	0	7.00
3	1	2.98 × 10^−3^	9.00 × 10^−4^	1.18 × 10^−3^	3.31
	2	6.18 × 10^−4^	2.36 × 10^−4^	1.67 × 10^−5^	2.62
	3	1.42 × 10^−4^	2.50 × 10^−5^	0	5.67
	4	0	0	0	*
	5	6.67 × 10^−5^	0	0	*
	6	6.67 × 10^−5^	8.33 × 10^−6^	0	8.00

* No large autosomal target DNA present to calculate DI.

**Table 2:** Spearman’s rank correlation coefficients (Spearman’s ρ) between weekly environmental factors and concentration of nuDNA derived from weekly soil samples, showing T-statistic and degrees of freedom (DF).

Relationship	Spearman’s ρ	T-Statistic	DF	*p*-Value
Week into Decomposition Period	−0.6594	3.5082	16	0.0029 *
TBS	−0.4977	2.2951	16	0.0356 *
Weekly Body Moisture Content	0.2668	1.1074	16	0.2845
Total Weekly Rainfall	0.1729	0.7023	16	0.4926
Weekly Average Humidity	−0.0167	0.0667	16	0.9477
Weekly Average Temperature	−0.6184	3.1479	16	0.0062 *

* Indicates a significant *p*-value (<0.05).

**Table 3:** Average Ct values and ΔΔCt calculations for soil samples from three donors across eleven weeks of surface-level decomposition using the Kavlick triplex mtDNA qPCR assay.

		Sample		Calibrator		
Donor	Week	Average Ct (105 bp)	Average Ct (316 bp)	Average Ct (105 bp)	Average Ct (316 bp)	ΔΔCt (Degradation)
1	1	27.93	35.10	24.66	30.10	1.73
	2	31.10	33.24	25.88	26.98	1.04
	3	27.37	30.99	24.15	30.27	−2.50
	4	31.75	33.51	25.32	26.52	0.56
	5	29.56	34.53	24.55	26.56	2.96
	6	30.62	33.25	25.73	26.98	1.38
	7	30.37	37.31	24.30	26.93	4.31
	8	29.81	39.02	21.03	26.80	3.44
	9	32.22	38.16	25.03	26.88	4.09
	10	33.94	38.72	26.31	28.12	2.97
	11	33.86	Undetermined	26.31	28.12	*
2	1	24.82	30.81	24.66	30.10	0.55
	2	27.12	28.66	25.88	26.98	0.44
	3	22.55	26.56	24.15	30.27	−2.11
	4	29.85	31.70	25.32	26.52	0.65
	5	29.62	33.89	24.55	26.56	2.26
	6	29.19	35.07	25.73	26.98	4.63
	7	29.50	35.68	24.30	26.93	3.55
	8	29.29	37.36	21.03	26.80	2.30
	9	30.98	35.16	25.03	26.88	2.33
	10	31.02	34.35	26.31	28.12	1.52
	11	31.18	37.70	26.31	28.12	4.71
3	1	25.70	32.93	24.66	30.10	1.79
	2	31.15	34.13	25.88	26.98	1.88
	3	30.15	37.02	24.15	30.27	0.75
	4	33.22	36.24	25.32	26.52	1.82
	5	29.66	35.04	24.55	26.56	3.37
	6	29.75	35.15	25.73	26.98	4.15
	7	29.13	36.80	24.30	26.93	5.04
	8	27.93	36.82	21.03	26.80	3.12
	9	30.35	34.46	25.03	26.88	2.26
	10	31.78	35.16	26.31	28.12	1.57
	11	30.74	35.43	26.31	28.12	2.88

* Indicates no large target was present to calculate ΔΔCt.

**Table 4:** Spearman’s rank correlation coefficients (Spearman’s ρ) between weekly environmental factors and copy number of mtDNA derived from weekly soil samples, showing T-statistic and degrees of freedom (DF).

Relationship	Spearman’s ρ	T-Statistic	DF	*p*-Value
Week into Decomposition Period	−0.6219	4.422	31	0.0001 *
TBS	−0.4687	2.9539	31	0.0059 *
Weekly Body Moisture Content	0.3274	1.9290	31	0.0629
Total Weekly Rainfall	0.1275	0.7160	31	0.4794
Weekly Average Humidity	−0.3439	2.0391	31	0.0500 *
Weekly Average Temperature	−0.7200	5.7759	31	2.3245 × 10^−6^ *

* Indicates a significant *p*-value (<0.05).

## Data Availability

The original data presented in the study are openly available in GitHub repository at https://github.com/HLNoel/Genes_DNA_Persistence/tree/main.

## Supplementary Materials

The following supporting information can be downloaded at: https://www.mdpi.com/article/10.3390/genes15060741/s1, Table S1: Quantifiler™ Trio qPCR replicate results on the quantity of small autosomal, large autosomal, and Y-chromosomal target nuDNA present in soil samples from six weeks of surface-level decomposition in ng/µL from Donor 1; Table S2: Quantifiler™ Trio qPCR replicate results on the quantity of small autosomal, large autosomal, and Y-chromosomal target nuDNA present in soil samples from six weeks of surface-level decomposition in ng/µL from Donor 2; Table S3: Quantifiler™ Trio qPCR replicate results on the quantity of small autosomal, large autosomal, and Y-chromosomal target nuDNA present in soil samples from six weeks of surface-level decomposition in ng/µL from Donor 3; Table S4: Data set used to calculate the effects of environmental factors on quantity of nuDNA (ng/µL); Table S5: Eigenvalues and percentage of variance explained by components for principal components analysis of environmental and decomposition factors influencing weekly soil samples following six weeks of surface-level decomposition, Table S6:Correlation coefficients for principal components analysis of environmental and decomposition factors influencing weekly soil samples following six weeks of surface-level decomposition, Table S7: Kavlick mtDNA multiplex qPCR replicate results on the quantity of mtDNA present in soil samples from eleven weeks of surface-level decomposition in copy number/µL from Donor 1; Table S8: Kavlick mtDNA multiplex qPCR replicate results on the quantity of mtDNA present in soil samples from eleven weeks of surface-level decomposition in copy number/µL from Donor 2; Table S9: Kavlick mtDNA multiplex qPCR replicate results on the quantity of mtDNA present in soil samples from eleven weeks of surface-level decomposition in copy number/µL from Donor 3; Table S10: Data set used to calculate the effects of environmental factors on quantity of mtDNA (copy number/µL); Table S11: Eigenvalues and percentage of variance explained by components for principal components analysis of environmental and decomposition factors influencing weekly soil samples following eleven weeks of surface-level decomposition, Table S12: Correlation coefficients for principal components analysis of environmental and decomposition factors influencing weekly soil samples following eleven weeks of surface-level decomposition.
