# Supplementary material for: Utilization of qPCR to Determine Duration and Environmental Drivers Contributing to the Persistence of Human DNA in Soil
Source: Genes (Basel). 2024 Jun 5;15(6):741. doi: 10.3390/genes15060741 (PMC11202937; doi:10.3390/genes15060741)
Supplement: Supplementary file 1 [file genes-15-00741-s001.zip › genes-3035271-supplementary.pdf]

Supplemental Table S1. Quantifiler™ Trio qPCR replicate results on the quantity of small autosomal, large autosomal, and Y-chromosomal target nuDNA present in soil samples from six weeks of surface level decomposition in ng/µl from **Donor 1.** C=Cranial, TR=Torso (Right Side), TL=Torso (Left Side), LE=Lower Extremity. Each following number corresponds to week into the decomposition period.

	Small Autosomal (ng/µl)			Large Autosomal (ng/ul)			Y-Chromosomal (ng/µl)		
**Sample**	**Rep 1**	**Rep 2**	**Rep 3**	**Rep 1**	**Rep 2**	**Rep 3**	**Rep 1**	**Rep 2**	**Rep 3**
**C-1**	0.0035	0.005*	0.0033	0.0011	0*	0.0017	0	0	0*
**TR-1**	0.0005	0	0.0001	0	0	0	0	0	0
**TL-1**	0.0004	0.0004	0.0003	0	0.0001	0	0	0	0
**LE-1**	0.0031	0.0036	0.0036	0.0001	0.0004	0.0002	0	0	0
**C-2**	0.0003	0.0003	0.0011*	0	0	0.0001*	0	0	0*
**TR-2**	0	0	0	0	0	0	0	0	0
**TL-2**	0.0002	0	0.0002	0	0	0.0001	0	0	0
**LE-2**	0.0002	0	0	0	0	0	0	0	0
**C-3**	0	0	0	0	0	0	0	0	0
**TR-3**	0	0	0	0	0	0	0	0	0
**TL-3**	0.0006	0.0006	0.0004	0	0	0	0	0	0
**LE-3**	0.0026*	0.0019	0.0018	0.0003*	0	0	0*	0	0
**C-4**	0	0	0	0	0.0002	0	0	0	0
**TR-4**	0	0	0	0	0.0002	0	0	0	0
**TL-4**	0	0	0	0	0	0	0	0	0
**LE-4**	0	0	0	0	0	0	0	0	0
**C-5**	0	0	0	0	0	0	0	0	0
**TR-5**	0.0006	0	0	0	0	0	0	0	0
**TL-5**	0	0	0	0	0	0	0	0	0
**LE-5**	0	0	0	0	0	0	0	0	0
**C-6**	0.0006	0	0	0	0	0.0001	0	0	0
**TR-6**	0.0004	0.0004	0	0	0	0	0	0	0
**TL-6**	0	0	0	0.0002	0	0	0	0	0
**LE-6**	0	0	0	0.0003	0	0	0	0	0

*Indicates removal based on Ct-SD when calculating average quantities.

Supplemental Table S2. Quantifiler™ Trio qPCR replicate results on the quantity of small autosomal, large autosomal, and Y-chromosomal target nuDNA present in soil samples from six weeks of surface level decomposition in ng/μl from **Donor 2.** C=Cranial, TR=Torso (Right Side), TL=Torso (Left Side), LE=Lower Extremity. Each following number corresponds to week into the decomposition period.

	Small Autosomal (ng/μl)			Large Autosomal (ng/ul)			Y-Chromosomal (ng/μl)		
**Sample**	**Rep 1**	**Rep 2**	**Rep 3**	**Rep 1**	**Rep 2**	**Rep 3**	**Rep 1**	**Rep 2**	**Rep 3**
**C-1**	0.0114	0.0092	0.0121	0	0.0001	0.0001	0	0.0004	0.001
**TR-1**	0.0003	0	0.0005	0.0003*	0	0	0.0001*	0.0006	0.0005
**TL-1**	0.0004*	0.0008	0.001	0.0026	0.003	0.003	0.0115	0.0083	0.0109
**LE-1**	0.0146	0.0128	0.0151	0.0126	0.0104	0.0093	0.0177	0.0138	0.0113
**C-2**	0.0008	0.0012	0.0023	0	0.0001	0*	0.0024	0	0.0009
**TR-2**	0.001	0.001	0.0017*	0	0.0002	0	0	0	0.0012
**TL-2**	0	0.0007	0	0	0	0	0	0	0
**LE-2**	0.0004	0.0008	0	0	0.001	0	0	0	0
**C-3**	0.0011	0.0006	0	0	0	0	0	0	0
**TR-3**	0.0006	0.0006	0	0	0	0	0	0	0
**TL-3**	0	0.0005	0	0	0	0	0	0	0
**LE-3**	0.0005	0.0006	0.0005	0	0	0	0	0	0
**C-4**	0	0	0	0	0	0	0	0	0
**TR-4**	0.0004	0	0	0.0002	0	0.0008	0	0	0
**TL-4**	0	0	0	0	0	0	0	0	0
**LE-4**	0	0	0	0	0	0	0	0	0
**C-5**	0	0.0007	0	0	0	0	0	0	0
**TR-5**	0.0004	0	0	0	0	0	0	0	0
**TL-5**	0	0	0	0	0	0	0	0	0
**LE-5**	0	0	0	0	0	0	0	0	0
**C-6**	0	0.0004	0.0004	0.0001	0	0.0001	0	0	0
**TR-6**	0	0.0003	0	0	0	0	0	0	0
**TL-6**	0	0	0	0	0	0	0	0	0
**LE-6**	0	0.0003	0	0	0	0	0	0	0

*Indicates removal based on Ct-SD when calculating average quantities.

Supplemental Table S3. Quantifiler™ Trio qPCR replicate results on the quantity of small autosomal, large autosomal, and Y-chromosomal target nuDNA present in soil samples from six weeks of surface level decomposition in ng/μl from **Donor 3.** C=Cranial, TR=Torso (Right Side), TL=Torso (Left Side), LE=Lower Extremity. Each following number corresponds to week into the decomposition period.

	**Small Autosomal (ng/μl)**			**Large Autosomal (ng/ul)**			**Y-Chromosomal (ng/μl)**		
**Sample**	**Rep 1**	**Rep 2**	**Rep 3**	**Rep 1**	**Rep 2**	**Rep 3**	**Rep 1**	**Rep 2**	**Rep 3**
**C-1**	0.0029	0.0021	0.0027	0	0.0002	0.0002	0.0005	0	0.0009
**TR-1**	0	0	0	0	0	0.0002	0	0	0
**TL-1**	0.0056	0.0056	0.0061	0.0003	0.0004	0.0001	0.0014	0.0005	0
**LE-1**	0.0029	0.0021	0.0027	0.0001	0	0.0093	0	0	0.0108
**C-2**	0	0	0	0	0	0	0	0	0
**TR-2**	0	0	0	0	0	0	0	0	0
**TL-2**	0.0013	0.0013	0.0007*	0	0	0*	0	0	0*
**LE-2**	0.002	0.002	0.0002	0.0005	0.0004	0.0017	0.0002	0	0
**C-3**	0.0007	0.0004	0	0	0	0	0	0	0
**TR-3**	0	0	0	0	0.0001	0	0	0	0
**TL-3**	0	0	0	0	0	0	0	0	0
**LE-3**	0	0	0	0	0.0002	0	0	0	0
**C-4**	0	0	0	0	0	0	0	0	0
**TR-4**	0	0	0	0	0	0	0	0	0
**TL-4**	0	0	0	0	0	0	0	0	0
**LE-4**	0	0	0	0	0	0	0	0	0
**C-5**	0	0	0	0	0	0	0	0	0
**TR-5**	0	0	0	0	0	0	0	0	0
**TL-5**	0	0	0.0008	0	0	0	0	0	0
**LE-5**	0	0	0	0	0	0	0	0	0
**C-6**	0.0002	0	0	0	0	0	0	0	0
**TR-6**	0	0.0003	0	0	0	0	0	0	0
**TL-6**	0	0	0	0	0	0	0	0	0
**LE-6**	0.0003	0	0	0	0.0001	0	0	0	0

*Indicates removal based on Ct-SD when calculating average quantities.

Supplemental Table S4. Data set used to calculate the effects of environmental factors on quantity of nuDNA (ng/μl). Environmental factors include time in weeks, Total Body Score (TBS), average weekly body moisture concentration (%), weekly total rainfall (cm), average weekly humidity, and average weekly temperature (Celsius).

Donor	Week	TBS	Body Moisture	Rainfall	Average Humidity	Average Temperature	Quantity nuDNA
1	1	23	45.43	1.2446	74.28571	17.37103	0.001709
	2	27	60	7.3406	80.14286	20.11905	0.000109
	3	31	33.77	5.588	78.85714	18.82937	0.000482
	4	31	37.56	1.2954	77.14286	19.3254	0
	5	30	40.57	0.1016	78.14286	21.47156	0.00005
	6	31	22.47	0.0762	67.42857	21.36905	0.000117
2	1	19	19.57	1.2446	76.33333	17.28009	0.007073
	2	24	60	7.3406	80.14286	20.11905	0.000745
	3	26	20.63	5.588	78.85714	18.82937	0.000417
	4	27	26.83	1.2954	77.14286	19.3254	3.33E-05
	5	28	19.47	0.1016	78.14286	21.47156	9.17E-05
	6	28	22.1	0.0762	67.42857	21.36905	0.000117
3	1	19	30.6	1.0922	76.16667	19.0625	0.002983
	2	22	30.5	1.2954	77.14286	19.3254	0.000618
	3	24	19.33	0.1016	78.14286	21.47156	9.17E-05
	4	24	17.6	0.0762	67.42857	21.36905	0
	5	24	18.33	2.1082	76	22.1131	6.67E-05
	6	26	22.4	1.7018	86.57143	22.24206	6.67E-05

Supplemental Table S5. Eigenvalues and percentage of variance explained by components determined from principal components analysis of environmental and decomposition factors influencing weekly soil samples following six weeks of surface level decomposition.

Component	Eigenvalue	Percentage of Variance
1	2.075	41.507
2	1.298	25.965
3	0.831	16.621
4	0.457	9.147
5	0.338	6.779

Supplemental Table S6. Correlation coefficients for principal components analysis of environmental and decomposition factors influencing weekly soil samples following six weeks of surface level decomposition.

Variable	Dimension 1 Correlation Coefficient	Dimension 2 Correlation Coefficient
TBS	-0.086	0.867
Body Moisture Content	0.812	0.186
Avg Weekly Rainfall	0.871	0.177
Avg Weekly Humidity	0.639	0.157
Avg Weekly Temperature	-0.491	0.675

Supplemental Table S7. Kavlick mtDNA multiplex qPCR replicate results on the quantity of mtDNA present in soil samples from eleven weeks of surface-level decomposition in copy number/µl from **Donor 1**.

	Cranium			Torso Right			Torso Left			Lower Extremity		
**Wk**	**Rep 1**	**Rep 2**	**Rep 3**	**Rep 1**	**Rep 2**	**Rep 3**	**Rep 1**	**Rep 2**	**Rep 3**	**Rep 1**	**Rep 2**	**Rep 3**
**1**	4955.7	2884.7*	4577.9	613.9	675.1	587.7	691.9*	462.5	412.2	328.9	312.9	465.7
**2**	254.1	356.3	303.4	234.5	243.3	258.3	1642.0	1413.3	1535.9	2329.6	2292.1	1011.1*
**3**	322.5	270.0	300.9	588.4	638.5	652.0	8118.3	8118.3	7552.3	6505.7	5898.9	6042.0
**4**	4496.3	4189.2	4473.1	342.1	408.6	446.7	479.7	417.9	434.5	328.0	332.1	343.4
**5**	229.3	226.7	207.0	3043.4	3218.6	3325.0	308.6	273.9	275.2	498.6	467.9	502.7
**6**	172.9	168.0	149.3	181.7	206.9	230.5	3284.8	3232.0	3110.5	5151.6	4787.6	5136.5
**7**	252.7	215.7	193.2	334.6	287.9	256.4	356.6	401.5	404.7	333.1	308.5	397.8
**8**	247.1	236.3	255.3	28.5	23.1	28.0	36.2	26.5	34.6	32.0	26.0	27.9
**9**	142.9	152.2	119.6	211.1	170.6	167.3	172.3	199.7	163.7	140.5	117.4	159.1
**10**	107.5	105.2	94.5	143.9	161.5	143.2	163.1*	103.0	123.1	71.0	86.4	107.5*
**11**	0	0	0	0	0	0	109.0	125.8	439.8	0	0	0

*Indicates removal based on Ct-SD when calculating average quantities.

Supplemental Table S8. Kavlick mtDNA multiplex qPCR replicate results on the quantity of mtDNA present in soil samples from eleven weeks of surface-level decomposition in copy number/µl from **Donor 2.**

	Cranium			Torso Right			Torso Left			Lower Extremity		
**Wk**	**Rep 1**	**Rep 2**	**Rep 3**	**Rep 1**	**Rep 2**	**Rep 3**	**Rep 1**	**Rep 2**	**Rep 3**	**Rep 1**	**Rep 2**	**Rep 3**
**1**	3577.0	4372.2	5686.0*	7458.5	6430.0	5802.0	4561.4	4817.2	7070.5*	10447.5	11545.0	7293.0
**2**	3592.9	3277.1	3676.9	12007.5	11076.5	11600.5	13287.0	13234.5	14265.5	14069.5	15139.0	18596.0
**3**	85328.5	78991.0	99925.5	57666.0	62896.0	65355.5	8540.5	8203.0	8551.4	148620.5	145307.0	137713.0
**4**	395.1	463.3	391.9	24647.5	15615.0*	24458.5	4567.2	4513.4	4188.7	1919.7	1780.7	1822.1
**5**	349.2	399.2	390.0	1176.8	1100.6	1206.7	422.3	415.7	455.7	434.2	431.4	463.8
**6**	2061.1	2313.9	2385.1	2338.7	2237.3	2101.0	2371.4	2396.7	2338.0	2147.4	2316.8	2372.7
**7**	422.5	487.1	533.7	568.7	718.3	514.4	584.9	546.8	663.2	510.6	667.4	666.6
**8**	81.0	79.5	100.4	62.8	283.4*	58.3	44.2	41.6	44.1	81.1	138.0*	75.4
**9**	220.0	249.2	256.8	366.2	329.5	349.2	518.3	479.9	556.5	300.9*	490.9	542.4
**10**	812.8	770.0	838.0	713.6	675.9	676.8	352.9	382.3	418.7	1931.5	2037.2	2104.4
**11**	0	0	0	0	0	0	784.1	668.7	732.0	0	0	0

*Indicates removal based on Ct-SD when calculating average quantities.

Supplemental Table S9. Kavlick mtDNA multiplex qPCR replicate results on the quantity of mtDNA present in soil samples from eleven weeks of surface-level decomposition in copy number/μl from **Donor 3.**

	Cranium			Torso Right			Torso Left			Lower Extremity		
**Wk**	**Rep 1**	**Rep 2**	**Rep 3**	**Rep 1**	**Rep 2**	**Rep 3**	**Rep 1**	**Rep 2**	**Rep 3**	**Rep 1**	**Rep 2**	**Rep 3**
**1**	1779.7	1872.4	2804.9*	388.4	618.8*	497.9	9694.5	9022.5	13754.5*	9759.5*	15893.5	20389.0
**2**	96.5	113.6	103.2	131.0	124.2	145.2	1365.5	1287.0	1303.7	8423.5	8146.0	7908.0
**3**	212.4	221.9	222.1	273.8	247.7	254.4	257.2	255.3	301.8	857.3	857.4	861.5
**4**	361.5	399.4	327.4	127.5	112.9	120.9	176.7	167.0	152.0	735.5	763.7	717.2
**5**	324.4	360.9	308.2	596.9	661.0	645.6	483.7	454.8	447.6	452.4*	923.1	932.7
**6**	2307.4	2230.4	2260.2	2317.7	2303.3	2364.8	2643.3	2498.3	2553.1	436.9	433.6	408.6
**7**	654.3	640.3	643.2	574.0	609.9	601.9	1027.6	1138.8	735.6*	821.3	809.2	871.0
**8**	59.1	48.0	93.6*	148.5	122.0	128.6	1743.9	3003.2*	1572.3	153.9	151.9	160.4
**9**	1184.3	1212.7	1126.0	915.3	889.4	923.7	1097.6*	809.9	727.7	68.2	113.7*	73.5
**10**	1502.2	1700.2	1689.7	551.4	549.9	562.7	408.6	320.5	365.3	166.5	179.3	154.9
**11**	1164.6	886.85	923.45	0	0	0	0	0	0	0	0	0

*Indicates removal based on Ct-SD when calculating average quantities.

Supplemental Table S10. Data set used to calculate the effects of environmental factors on quantity of mtDNA (copy number/µl). Environmental factors include time in weeks, Total Body Score (TBS), average weekly body moisture concentration (%), weekly total rainfall (cm), average weekly humidity, and average weekly temperature (Celsius).

Donor	Week	TBS	Body Moisture	Rainfall	Average Humidity	Average Temperature	Quantity mtDNA
1	1	23	45.43	1.2446	74.28571	17.37103	2872.62
	2	27	60	7.3406	80.14286	20.11905	1975.046
	3	31	33.77	5.588	78.85714	18.82937	7488.824
	4	31	37.56	1.2954	77.14286	19.3254	2781.91
	5	30	40.57	0.1016	78.14286	21.47156	2096.136
	6	31	22.47	0.0762	67.42857	21.36905	4302.036
	7	31	27.2	2.1082	76	22.1131	623.7694
	8	31	30.6	1.7018	86.57143	22.24206	166.9407
	9	32	60	2.1844	85.28571	22.21561	319.4035
	10	32	0	0.4826	82.42857	21.81548	227.8508
	11	32	0	0.9652	82	23.13492	234.8125
2	1	19	19.57	1.2446	76.33333	17.28009	13113.38
	2	24	60	7.3406	80.14286	20.11905	22303.75
	3	26	20.63	5.5880	78.85714	18.82937	151183
	4	27	26.83	1.2954	77.14286	19.3254	12572.36
	5	28	19.47	0.1016	78.14286	21.47156	1207.61
	6	28	22.1	0.0762	67.42857	21.36905	4563.297
	7	28	25.6	2.1082	76	22.1131	1147.311
	8	28	25.23	1.7018	86.57143	22.24206	133.6709
	9	28	22.2	2.1844	85.28571	22.21561	792.4656
	10	28	23.87	0.4826	82.42857	21.81548	1952.309
	11	30	19.4	0.9652	82	23.13492	1456.52
3	1	19	30.6	1.0922	76.16667	19.0625	14884.47
	2	22	30.5	1.2954	77.14286	19.3254	4857.852
	3	24	19.33	0.1016	78.14286	21.47156	803.8077
	4	24	17.6	0.0762	67.42857	21.36905	693.6028
	5	24	18.33	2.1082	76	22.1131	1116.144
	6	26	22.4	1.7018	86.57143	22.24206	3792.903
	7	28	16.93	2.1844	85.28571	22.21561	1525.67
	8	28	21	0.4826	82.42857	21.81548	857.6823
	9	28	20.03	0.9652	82	23.13492	1586.097
	10	29	27.37	3.0226	87.57143	22.18254	1358.518
	11	29	30.97	2.2606	88.57143	21.33929	1983.269

Supplemental Table S11. Eigenvalues and percentage of variance explained by components determined from principal components analysis of environmental and decomposition factors influencing weekly soil samples following eleven weeks of surface level decomposition.

Component	Eigenvalue	Percentage of Variance
1	2.017	40.334
2	1.562	31.232
3	0.682	13.645
4	0.416	8.313
5	0.324	6.477

Supplemental Table S12. Correlation coefficients for principal components analysis of environmental and decomposition factors influencing weekly soil samples following eleven weeks of surface level decomposition.

Variable	Dimension 1 Correlation Coefficient	Dimension 2 Correlation Coefficient
TBS	0.671	0.435
Body Moisture Content	-0.625	0.568
Avg Weekly Rainfall	-0.524	0.717
Avg Weekly Humidity	0.374	0.703
Avg Weekly Temperature	0.873	0.201
